# Thinking globally, acting locally in the 21^st^ century: Bamboo to bioproducts and cleaned mine sites

**DOI:** 10.1016/j.isci.2024.110763

**Published:** 2024-09-07

**Authors:** Michael T. Timko, Timothy M. Woodard, Aubrey E. Graham, Julian A. Bennett, Robert Krueger, Aidin Panahi, Nima Rahbar, James Walters, Darnell Dunn

**Affiliations:** 1 Department of Chemical Engineering, Worcester Polytechnic Institute, 100 Institute Road, Worcester, MA 01609, USA; 2 Department of Chemistry & Biology, Worcester Polytechnic Institute, 100 Institute Road, Worcester, MA 01609, USA; 3 Department of Social Science & Policy Studies, Worcester Polytechnic Institute, 100 Institute Road, Worcester, MA 01609, USA; 4 Department of Civil, Environmental, & Architectural Engineering, Worcester Polytechnic Institute, 100 Institute Road, Worcester, MA 01609, USA; 5 Institute of Science & Technology for Development, Worcester Polytechnic Institute, 100 Institute Road, Worcester, MA 01609, USA; 6 Avos Bioenergy, 3187 Danmark Dr, West Friendship, MD 21794, USA; 7 School of Business, Worcester Polytechnic Institute, 100 Institute Road, Worcester, MA 01609, USA

**Keywords:** Chemical engineering, Biotechnology, Biomass, Engineering

## Abstract

Current solutions to global challenges place tension between global benefits and local impacts. The result is increasing opposition to implementation of beneficial climate policies. Prioritizing investment in projects with tangible local benefits that also contribute to global climate change can resolve this tension and make local communities’ partners instead of antagonists to change; the approach advocated is a new take on “thinking globally, acting locally”. This approach is a departure from the usual strategy of focusing resources on solutions perceived to have the largest potential global impact, without regards to local concerns. Reclamation of polluted mine sites by using fast growing bamboo to remove heavy metals provides a case study to show what is possible. Effective implementation of thinking globally while acting locally will require increased coordination between different types of researchers, new educational models, and greater stakeholder participation in problem identification and solution development.

## Graphical abstract

**Figure:**
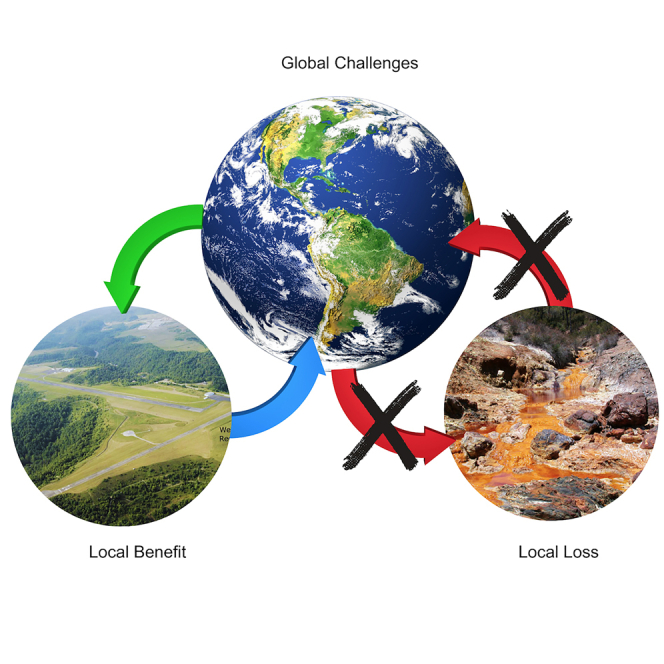

## Thinking globally

Mass migration, climate change, pandemics, and many of the other threats and challenges of the 21^st^ century are inherently global in nature. Global challenges call for concerted and consistent effort worldwide, which is the impetus behind the 17 Sustainable Development Goals laid out by the United Nations. At the same time, global challenges are diffuse and have distributed impacts that are seldom localized at the community, household, and individual levels.^1^ Moreover, solving, or mitigating problems, such as global pandemics, is difficult to appreciate—how does one quantify the benefit of preventing a disaster or reducing a threat that never occurred?^2^

Global climate change has been on the political agenda for nearly 50 years.^3^ Scientific consensus agrees that the Earth has been getting hotter. Science has now indisputably demonstrated the causality between climate change and increasingly powerful storms, changes in ocean temperature and currents that lead to as-of-yet unpredictable weather patterns, and ocean acidification that damages the aquatic ecosystems that support most of life on Earth.^4^ Despite this scientific consensus, finding the political will to curb greenhouse gas (GHG) emissions remains elusive, with specific pushback against projects with perceived negative localized impacts and less tangible global benefits.^5^ The abstract nature of global threats, perhaps especially climate change, gives rise in part to the polarization we have seen; those who prioritize immediate benefits and fear concrete threats over potentially enormous, yet more abstract ones, cannot be dissuaded by the logic of those who believe the opposite. In many instances, and in locations all around the world, the result is a stalemate—and a stalemate is a luxury the world cannot afford.

When it comes to the places where we live and do business, however, everyone becomes an environmentalist—or, at least, they are more environmentally motivated by local issues than global ones. Nobody wants to live in a toxic environment, expose themselves or their children to pollution, or face a future with the risk of disease associated with living with polluted air, water, or soil. Concern for themselves and their families is why, whenever possible, those with economic means escape unclean environments. This effect is starkly evidenced by the demographics of the “Cancer Alley” of Louisiana compared with the rest of that state and Southeastern Texas.^6^ An invisible, gradual, and potentially global threat is far more difficult to understand than a threat in one’s backyard.^7^ This observation further explains the pushback, even from self-professed environmentalists, regarding renewable energy projects in their neighborhoods (e.g., Cape Wind).^8^

Mitigating local environmental damage requires changes in practice. In the U.S., adopting Best Available Control Technology (BACT) reduces chemical factory emissions, but it comes at a cost.^9^ Perversely, many of the renewable energy technologies upon which we pin our hopes require mining precious or earth-scarce materials in locations all across the globe.^10^ Yet, mined lands are a blight to their host economies, and reclaiming contaminated mining sites requires enormous sums of money—often from companies that have been bankrupt for a generation.^11^ For fossil fuels, a prime contributor to climate change, only the weakest of regulations, such as Corporate Average Fuel Economy (CAFE) standards, are in place. Recent CAFE standards announced by the climate-forward Biden Administration would reduce fuel needs by 200 billion gallons, which is roughly equivalent to what our entire country uses in just 60 days.^12^ While this is a positive step toward better fuel efficiency for all of us, 60 days of annual fuel usage is just a drop in the bucket compared to the massive changes needed. Is there an unresolvable tension between local and global threats?

Here, we propose an approach to identifying situations in which addressing local problems can bring local benefits, while also contributing solutions to global challenges. Naturally, we concede that this approach may not apply in every scenario. Some regional problems will defy solutions that lead to measurable global benefits, and, regrettably, vice versa. However, when situations that fall within this categorization are identified, they should be prioritized, so that the cumulative effect can achieve the local benefits and global impacts needed.

We propose a modified version of the old slogan, “Think globally, Act locally.” While the original intention of this slogan was one of personal responsibility to encourage consumers to conserve electricity (e.g., by turning off unused lights or recycling), the local actions we have in mind are on a much larger scale.^13^ We seek local problems that, when solved, will bring local benefits—such as cleaner environments and economic development.

We recognize that our proposal has a common cause with the concept of “co-benefits”, a term generally used to describe benefits arising from policy changes, in addition to any that may help mitigate climate change.^14^ Distinguishing our perspective from co-benefits is our emphasis on (a) expanding co-benefits to include solutions that address problems other than climate change, while giving rise to both local and global benefits, (b) focusing more heavily on the localized benefits that individuals and communities can easily recognize, and (c) emphasizing the positive role of science, especially the chemical sciences, in solving environmental problems. Examples include: science-informed actions that benefit the health of communities, while minimizing potential for emergence of new pandemics; local conservation methods that benefit economic opportunities while maintaining global biodiversity; and, as is the focus of this perspective, activities that benefit local environment and economy while reducing GHG emissions to mitigate climate change.

While the approach suggested may seem to be overly optimistic, perhaps even naive, we present one concrete example of the methodology, with the understanding that other appropriate contexts exist, especially at the intersection of academic disciplines and in industries found worldwide. As a global industry that relies on a variety of science technologies, mining (and the methods for remediation in its aftermath), provides an ideal opportunity to think locally and act globally. We advance our idea so that researchers from all backgrounds may apply our method to identify further opportunities for thinking globally and acting locally.

## Reclaiming abandoned mine sites as a case study

The U.S. Bureau of Land Management estimates that half a million abandoned mine sites cover more than 850,000 acres of land, with another 1.3 million acres of active mines on federal lands.^15^^,^^16^ Nearly all this land needs or will need extensive rehabilitation to make it fit for social and ecosystem benefits. The challenge is that the soil on these sites is contaminated by the acid drainage and associated heavy metal pollution generated by decades of mining.^17^ Mining contracts include clauses for restoration, but the money and bonds set aside are never sufficient for reclamation, especially when mining companies often declare bankruptcy, and their creditors are prioritized over environmental fines. The victim is the local population, whose mining jobs have been lost and whose local environments are seemingly irreversibly polluted.^18^

One promising method for reclaiming mine sites contaminated with heavy metals is phytoremediation, which leverages the uptake of heavy metals by plants.^19^ Plants naturally take up nutrients, including metals, from the soil through a combination of physiological and molecular processes, an activity aided by the relationship between plants and mycorrhizal fungi.^20^^,^^21^ Heavy metals accumulate in plant tissues and then in organisms further up the food chain when the plants are consumed.^20^ Phytoremediation capitalizes on a plant’s natural absorption capabilities by having plants take up undesirable metals left in the soil after mining and then disposing of the plants before the metals can enter the food system.^19^

**Figure 1:**
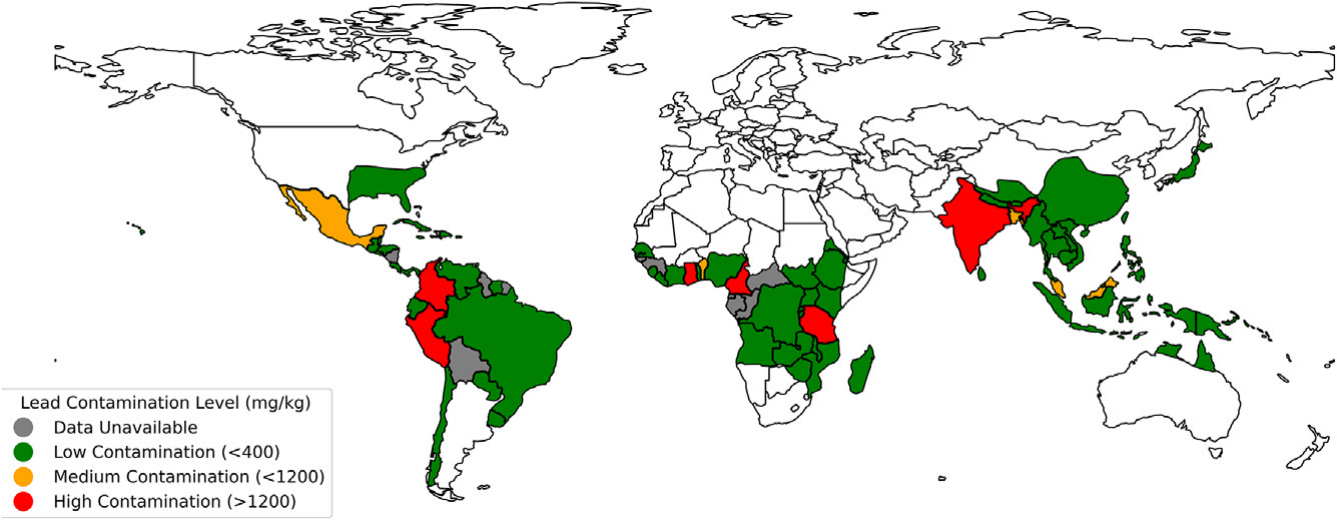
Worldwide distribution of bamboo growth regions highlighted with a heatmap showing lead contamination levels in the topsoil utilizing available data Sources cited in the text.^28^^,^^29^^,^^30^^,^^31^^,^^32^^,^^33^^,^^34^^,^^35^^,^^36^^,^^37^^,^^38^^,^^39^^,^^40^^,^^41^^,^^42^^,^^43^^,^^44^^,^^45^^,^^46^^,^^47^^,^^48^^,^^49^^,^^50^^,^^51^^,^^52^^,^^53^^,^^54^^,^^55^^,^^56^^,^^57^^,^^58^^,^^59^^,^^60^^,^^61^^,^^62^^,^^63^^,^^64^^,^^65^^,^^66^^,^^67^^,^^68^^,^^69^^,^^70^^,^^71^^,^^72^^,^^73^^,^^74^^,^^75^^,^^76^^,^^77^^,^^78^^,^^79^^,^^80^^,^^81^^,^^82^^,^^83^^,^^84^^,^^85^^,^^86^^,^^87^^,^^88^^,^^89^^,^^90^^,^^91^^,^^92^^,^^93^^,^^94^^,^^95^^,^^96^^,^^97^^,^^98^^,^^99^^,^^100^

The ideal plant for phytoremediation has several characteristics: (1) it must have the propensity to uptake heavy metals (ideally, as a hyperaccumulator) and have a high tolerance for soil contaminants; (2) it must exhibit rapid growth on marginal soil, with minimal inputs such as water or fertilizer; (3) it should resist local pests, be hardy to the local climate, and, when possible, be a native species; (4) it should provide additional benefits aside from heavy metal removal.^22^ One plant—or rather a group of plants—that fulfills all these roles defined by the ideal phytoremediation characteristics is bamboo. Bamboo comprises over 1,500 species and is native to many tropical and subtropical environments in the world, as illustrated with the highlighted growth regions in Figure 1.^23^^,^^24^ Bamboo is fast-growing (with growth rates ten times greater than corn), is suitable for marginal land, and requires minimal inputs.^25^ A broad range of bamboo species have been evaluated for phytoremediation with promising results in both laboratory-based and field studies for the accumulation of toxic heavy metals (e.g., Pd, Cd, Zn, Cr, Cu, and Mn) from soil.^26^ As an example of the potential utilization of bamboo, Figure 1 portrays both the expanses where bamboo flourishes and the lead contamination level in those regions’ topsoil. As previously mentioned, among the numerous abandoned mines across the United States, an estimated 633,000 acres are situated in the Appalachian region—an area coinciding with suitable growth regions for bamboo.^27^ Harnessing bamboo’s phytoremediation prowess toward those abandoned mine sites holds promise in mitigating these contamination levels, potentially offering a solution to environmental concerns if appropriately utilized.

Bamboo’s natural diversity allows for species selection based on a specific set of traits relevant to ideal phytoremediation characteristics.^101^ Certain timber bamboo species, such as *Phyllostachys* (*vivax*, *edulis*, *bambusoides*, *nuda*, *dulcis*, *rubrimarginata*, and *aurea*), exhibit rapid growth potential, controllable using various management techniques. *Arundinaria gigantea*, a bamboo species native to the Southeastern U.S., spreads more slowly but is rigorous as a sediment runoff buffer and a soil conditioner.^102^ Timber bamboo, particularly *Phyllostachys*, is especially promising for soil transformation and biodiversity enhancement.^103^ Simultaneously, timber bamboo’s tall canes create vertical diversity, promoting biodiversity by providing habitats for insects and small animals.^104^ Temperate *Bambusa*, including *Bambusa vulgaris*, offer remarkable anchoring abilities, well-suited for containing running bamboo. They achieve this through robust root systems that spread more vertically than horizontally, effectively anchoring soil to prevent erosion.^105^ Additionally, temperate *Bambusa* have a clumping growth habit, simplifying containment within designated areas and preventing the bamboo from becoming invasive. When planted strategically, these bamboo species act as natural barriers, reducing landslides and erosion risks during heavy rainfall.^106^

Compositionally, the dry matter of bamboo is composed primarily of lignin, cellulose, and hemicellulose. The cellulose and hemicellulose biopolymers within bamboo can be depolymerized to produce simple sugars, which are the building blocks for many valuable fuel and chemical products.^107^ Lignin can be converted into char and used for carbon sequestration either on the mining site or elsewhere.^108^ Alternatively, bamboo can be used as a construction material.^109^ Physicochemical treatments of bamboo or its components yield materials with even more promising mechanical properties than the plant itself.^110^ Depending on the harvest time, bamboo exists as a cellulose-rich plant that is ideal for depolymerization to produce simple sugars or a more lignin-rich version with more favorable mechanical properties.^111^

**Figure 2:**
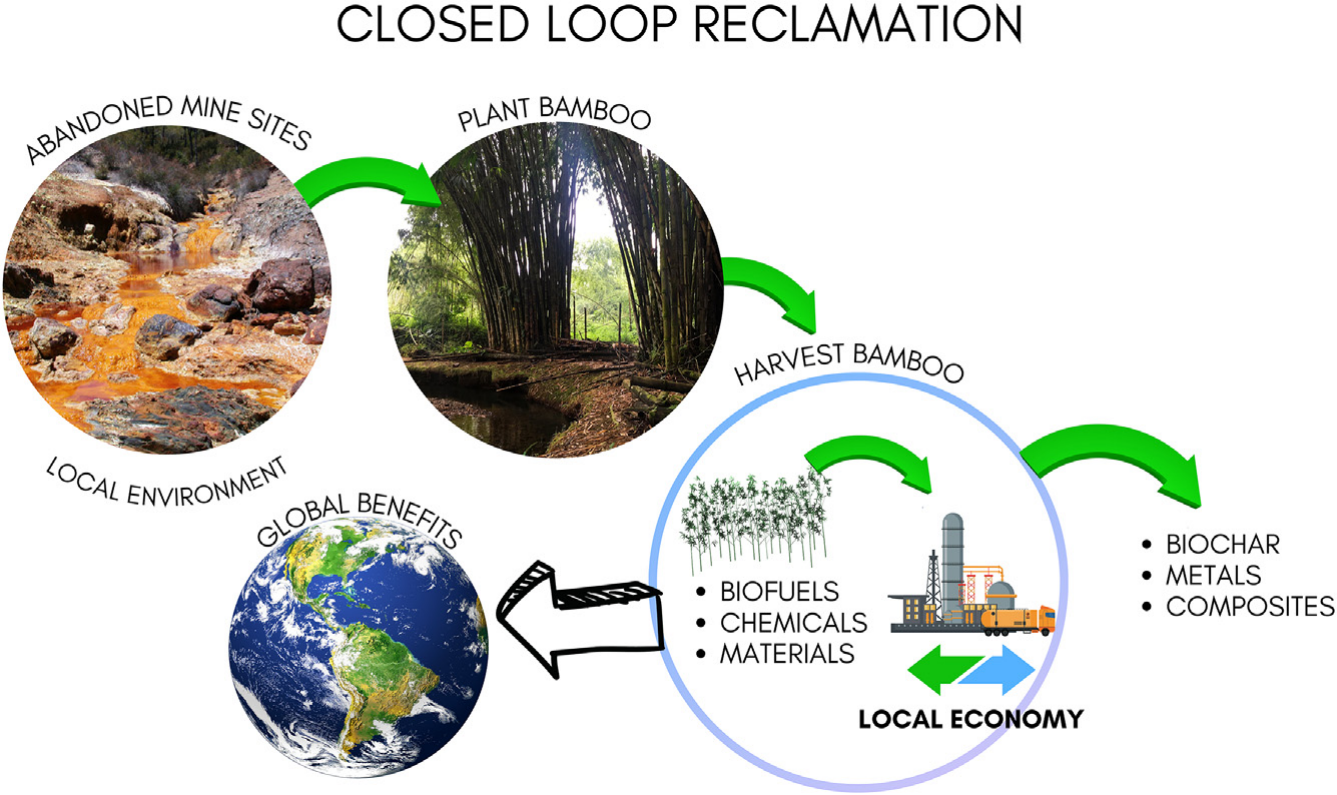
Bamboo can be grown on contaminated mine sites, extracting heavy metals to restore the land to a usable area Bamboo can also be harvested for a wide range of low-carbon products, including biofuels, building materials, biochar, and bio-based chemicals. Individual images in this composite were obtained from used with fair use permission from Canva.

Using biomass to produce construction materials is an ancient custom, and converting biomass to fuels has been studied on a small scale for decades.^112^ What we argue is that reclaimed mine sites—along with decarbonized fuels, chemicals, and materials—be included as a “product” of the bamboo process. An idealized schematic (Figure 2) illustrates the envisioned use of bamboo to decontaminate mine sites while simultaneously producing valuable products that reduce global emissions.^113^ Chemical process selection for conversion of bamboo into bioproducts depends on the application, with the selection process reflecting local concerns and traditions, in addition to optimizing economic value. Conversion technologies must be efficient and low-waste, prioritizing energy-efficient and atom-efficient processes that use minimal auxiliary chemicals. The generation of toxic wastes or byproducts must be avoided, and further research is required into the separation of usable end-products from contaminated biomass. The intended local environmental benefits cannot be realized otherwise.

**Figure 3:**
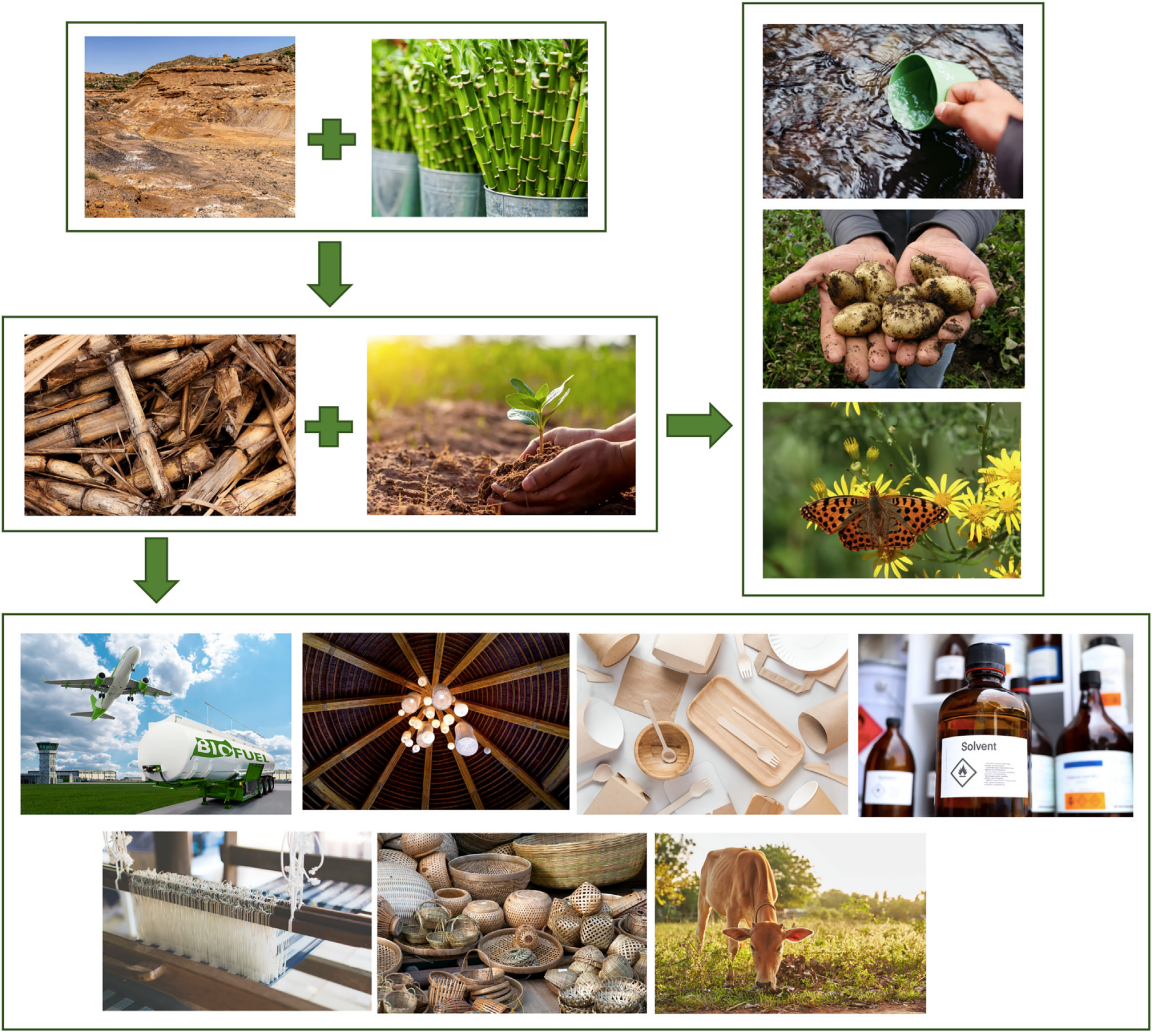
Bamboo for remediation regenerates mined lands with numerous positive effects (reforestation, improved water and air quality, carbon sequestration, and healthier soils and food products) while providing the source for more sustainable products (fuels, building materials, pulp and paper, bio-based chemicals, textiles, handicrafts, and livestock feed) that can revitalize economies Individual images in this composite were obtained from Canva or used with fair use permission from Adobe.

Technology selection should allow the local population to accrue as many financial benefits as possible. The economic benefits generated must support local labor markets and ancillary needs.^114^ The diversity and versatility of bamboo end products as biofuel feedstock, construction materials, fiber, paper pulp, handicrafts, water pipes, bicycles, livestock feed, and more (illustrated in Figure 3),^113^ empower communities to tailor products to the market and offer further business opportunities. The cultivation, processing, and refining of bamboo locally allows for economic growth and enables communities to benefit from cheaper, low-carbon energy.

Using native bamboo species for soil remediation and phytoremediation draws justification from historical precedents, species selection, and environmental benefits. In the Southeastern U.S., European settlers acknowledged the exceptional soil quality within bamboo canebrakes, making them prime candidates for agriculture and livestock forage.^102^ Native Americans utilized controlled burns to optimize these bamboo-rich lands, accelerating bamboo regrowth and using charred bamboo for soil conditioning, exemplifying resource harmonization through indigenous practices.^115^ Current agricultural and land-use practices break with these time-honored traditions to maximize short-term productivity. Bamboo growth on mine sites, where agriculture is no longer feasible, offers an opportunity to return to these practices.

The economics of converting bamboo to bioproducts benefits significantly from the localized environmental impacts we aim to achieve. Abandoned mine sites are often available at near-zero prices since the sites cannot be used for other applications.^116^ And, in many cases, funds have been set aside for mine site reclamation.^117^ Unfortunately, these funds are often insufficient for the task, and the company assigned to the cleanup frequently becomes insolvent before completing the job.^118^ By combining reclamation activities with the generation of a viable product or products—in this case, a bamboo-derived bioproduct, the economics can be favorable and reduce the risk of insolvency before the reclamation project is complete.

Selection of an optimal bamboo-derived product is key to achieving favorable economic performance and potential global benefits. The clear objective for global benefits is GHG emissions reduction, which is desperately needed to reduce global warming to manageable levels.^119^ Case-by-case holistic analysis that takes into account local factors is necessary for optimal product selection. Many product options are available, some of which include, use as low-carbon construction materials,^120^^,^^121^ torrefaction to biochar to ammend soil quality,^122^ or production of fuels or chemicals.^123^^,^^124^ Recent analysis indicates that bamboo-derived ethanol and simple sugars can be produced economically with available conversion technologies, even under economically unfavorable circumstances, for corn stover, long the favored feedstock of U.S.-based cellulosic fuels enthusiasts.^107^^,^^125^ More importantly, whereas the emissions reduction potential of corn-derived ethanol is debatable,^126^ the emissions reduction potential of second generation ethanol derived from biomass^127^^,^^128^ is estimated to be significant, and other second generation biofuels similar to ethanol may generate even fewer emissions than electrification.^129^

Achieving economic viability for biomass-derived ethanol is a long-standing challenge that bamboo utilization has the potential to overcome.^130^ Although several properties play a role in the economic feasibility of bamboo-derived fuels, the most important is its prodigious growth rate, which permits biorefinery operation at large and favorable scales, while managing transportation costs. The economic rationale supporting bamboo’s role in phytoremediation and the restoration of mine sites gains substantial traction when coupled with cost-effective land and a suite of monetary incentives, including carbon credits, tax incentives, and government grants associated with cleaning up the sites.^131^

The bamboo-to-bioproduct approach to mine reclamation can be duplicated in places around the world, from Appalachia in the U.S. to the gold mines of Ghana.^132^^,^^133^ Site restoration requires patience and may take decades for complete remediation; however, these sites will remain contaminated for centuries without a targeted effort.^134^ Economic benefits and local job opportunities will arrive much earlier in the project timeline than the full environmental benefits.

No global challenge can be resolved using only one technique; there are some locations that may be too dry, cold, or remote for the phytoremediation approach to be feasible. For these sites, other approaches will be necessary. However, a remediation-to-product model demonstrates the type of global thinking and local action necessary to tackle these challenges. To that end, the fate of heavy metal contaminants must be clearly understood for successful reclamation.

Other concerns must be addressed. The chemical processing of bamboo that we advocate here will remove heavy metals from the mine site without changing their form. Electrochemical methods, which involve the directed migration of heavy metal ions toward an electrode surface, selectively removing and depositing them to purify water streams from contaminants, then can be utilized on subsequent concentrated heavy metal streams to convert them into less naturally bioavailable forms.^135^ Equally important for any solution that seeks to benefit local development and achieve global impact is a focus on how technologies and solutions are deployed in the communities they serve. Open lines of communication must be achieved and maintained with all relevant stakeholders to ensure local acceptance. An understanding of local culture and context, the use of the products, and the economy of the place is required. Designing *for* a community is insufficient; only by designing *with* each community will the desired progress be achieved for a sustainable, global benefit.

## The role of (chemical) sciences

An updated “think globally, act locally” concept should inspire chemical scientists to think differently and perhaps more holistically about problem-solving and technology development. Historically, the main (though not exclusive) stakeholders in chemical science have been petrochemical and pharmaceutical companies. Outreach to local communities has been left to marketing, sustainability, and other departments far removed from the day-to-day of research and development. Considering local communities as stakeholders earlier in the pipeline is entirely different conceptually in terms of the types of problems to be solved, the scope of acceptable solutions, and the available resources.

Of course, the fundamentals of reactions, phase behavior, and other chemical transformations and processes are independent of context. However, how these fundamentals manifest themselves and even what questions we ask depend on context. For example, binding between biologically active metals, such as zinc, in the human body far outpaces study of the interactions and hence uptake of heavy metals into plant material. Heavy metal uptake is best understood as a chelating phenomenon that, if studied with the same vigor that similar problems in drug development have been pursued, could lead to tremendous breakthroughs in heavy metal uptake. That stated, technology development driven entirely by performance is inappropriate for thinking globally and acting locally. Even economic drivers, on their own, are insufficient. Instead, chemical scientists must learn to think with many different stakeholders in mind simultaneously, from local landowners and municipal agencies to investors.

Such interdisciplinary thinking allows for greater growth and creativity than may be found in a singular field and for the mining of inspiration from a broader range of sources. For example, integrating modern A.I., machine learning, and soil sensors with bamboo cultivation can optimize productivity and soil remediation. By combining the time-honored practices of indigenous communities with modern industrial operational optimization, the full potential of perennial closed-loop reclamation could create a foundational legacy of ecological sustainability for future generations.

Chemical solutions will undoubtedly differ when the range of stakeholders is expanded beyond established industries that have historically been the main beneficiaries of new chemical technologies. At a minimum, the utilization of waste will be prioritized, waste generation will be minimized, locally valued products will be emphasized, local cultural practices and preferences will be respected, and the use of local components and talent will be maximized. As much as possible, thinking about and planning for the impact of new chemical technologies—both positive and negative—on the full range of stakeholders will be critical in creating sustainable solutions.

## Parting thoughts

**Figure 4:**
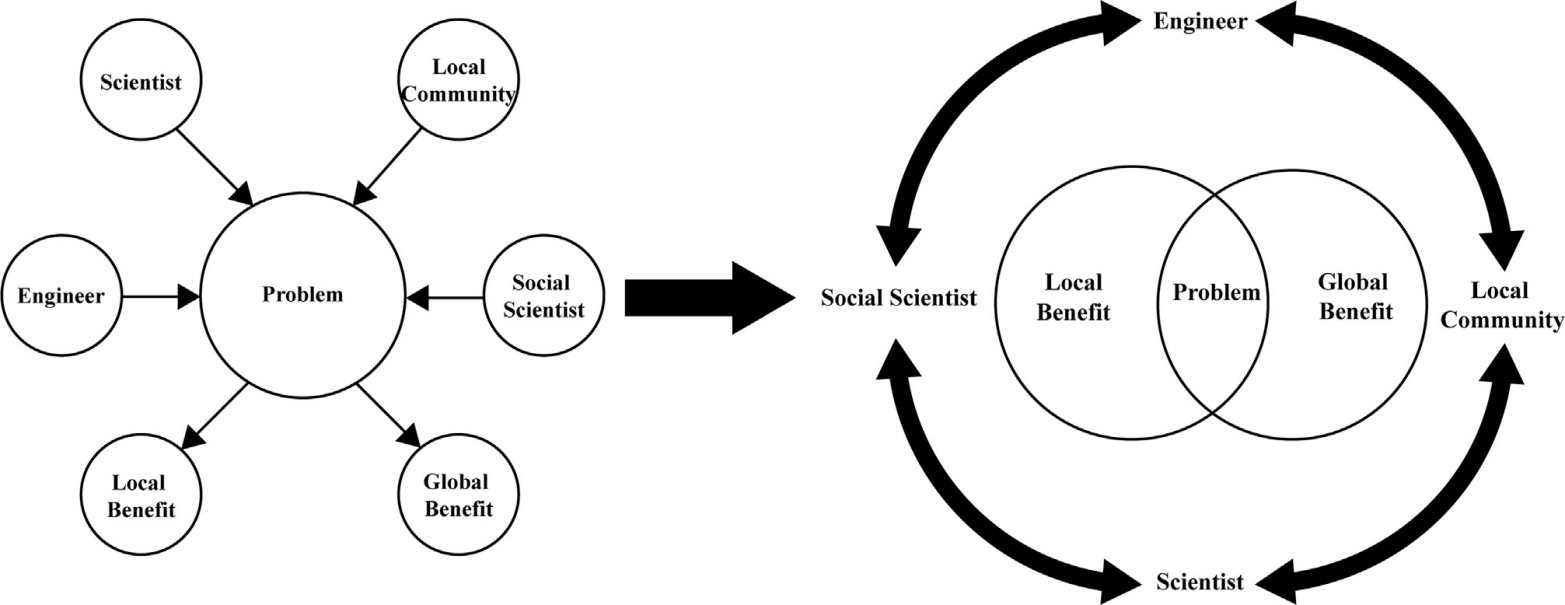
The current approach of collaboration on a specific global problem compared to a more collaborative approach in which cooperation is established before the problem is tackled resulting in a more directed local and global benefit

Chemical scientists of all types can contribute to local action inspired by global challenges. To bring truly transformative solutions, chemical scientists must work increasingly with other disciplines—physical and life sciences, engineering, social sciences, and the humanities. As such, Figure 4 was designed to illustrate the needed shift from the conventional approach of different disciplines contributing to a global problem independently to a more collaborative approach, where different disciplines are more intertwined in cooperation before even tackling the problem.

There are challenges to this level of collaboration, chiefly barriers associated with differences in jargon and incentive schemes that often value individual excellence in increasingly narrow areas of specialization, rather than broad expertise that leads to impactful, or potentially impactful, problem-solving. New educational paradigms that incentivize interdisciplinary training without sacrificing disciplinary rigor will be required. Addressing these challenges is imperative if science is to have a desirable influence on everyday life, while averting the worst global-scale catastrophes that science predicts will occur.

Acting locally while using an interdisciplinary model allows us to realize the untapped potential of underrepresented voices and overlooked regions. Embracing diversity in scientific collaboration, both in terms of people and geographic locations, can unearth unique insights and solutions that might have been overlooked in more homogeneous contexts. Thinking globally while acting locally becomes feasible only when actively seeking out and valuing contributions from all corners of the globe to unlock the global brainpower needed to confront the challenges that lie ahead.

### Limitations of the study

The theoretical case study presented faces societal, governmental, economic, and technical limitations. Navigating existing regulations, procedures, and funding to make local solutions broadly accessible is a significant challenge. Global problems are often seen as the domain of regulatory bodies and corporations, while local issues are handled by small businesses and individuals. A shift in perception is necessary to facilitate collaboration between stakeholders at different levels and to promote solutions that bring tangible local benefit, while contributing to solution of global problems. More specifically to the case study, more research is needed for selection of bamboo species and cultivation practices to optimize growth rate, metal uptake, bioproduct potential, and suitability for a given climate and economy. Finally, mined lands often contain several types of contamination, and in some cases, hyperaccumulating plants other than or in addition to bamboo might be required.

## Acknowledgments

T.M.W. and M.T.T.’s contributions were supported by the U.S. 10.13039/100000001National Science Foundation (#2021871).

## Author contributions

M.T.T. conceptualization, writing—original draft, supervision. T.M.W. writing—review and editing, formal analysis, data curation, visualization. A.E.G. writing—original draft, writing—review and editing, visualization. J.A.B. writing—review and editing. R.K. conceptualization, supervision, writing—original draft, writing—review and editing. A.P. writing—review and editing. N.R. writing—review and editing. J.W. conceptualization, writing—original draft, and visualization. D.D. writing—review and editing.

## Declaration of interests

The authors declare no competing financial interests.

## References

[1] United Nations (2022). The 17 Goals. https://sdgs.un.org/goals.

[2] Stoddard I., Anderson K., Capstick S., Carton W., Depledge J., Facer K., Gough C., Hache F., Hoolohan C., Hultman M. (2021). Three Decades of Climate Mitigation: Why Haven't We Bent the Global Emissions Curve?. Annu. Rev. Environ. Resour..

[3] Weart S.R. (2009). The idea of anthropogenic global climate change in the 20th century. WIREs Climate Change.

[4] (2023). How Do We Know Climate Change Is Real?. https://climate.nasa.gov/evidence/.

[5] Lesnikowski A., Biesbroek R., Ford J.D., Berrang-Ford L. (2021). Policy implementation styles and local governments: the case of climate change adaptation. Environ. Polit..

[6] Meaders J.S. (2021). Health Impacts of Petrochemical Expansion in Louisiana and Realistic Options for Affected Communities. Tulane Environ. Law J..

[7] Terrell K.A., St Julien G. (2022). Air pollution is linked to higher cancer rates among black or impoverished communities in Louisiana. Environ. Res. Lett..

[8] Gonyo S.B., Fleming C.S., Freitag A., Goedeke T.L. (2021). Resident perceptions of local offshore wind energy development: Modeling efforts to improve participatory processes. Energy Pol..

[9] (2023). New Source Performance Standards for the Synthetic Organic Chemical Manufacturing Industry and National Emission Standards for Hazardous Air Pollutants for the Synthetic Organic Chemical Manufacturing Industry and Group I & II Polymers and Resins Industry. Fed. Regist..

[10] Ojiambo M.N., Adachi T. (2023). Selected critical metals for a low-carbon future. Miner. Econ..

[11] Scott Pham K.W.J., Jacobs J. (2023). How We Measured the Environmental Cost of Bankrupt Mines. https://www.propublica.org/article/how-we-measured-environmental-cost-bankrupt-mines.

[12] (2022). USDOT Announces New Vehicle Fuel Economy Standards for Model Year 2024-2026.

[13] Shrivastava P., Stafford Smith M., O’Brien K., Zsolnai L. (2020). Transforming Sustainability Science to Generate Positive Social and Environmental Change Globally. One Earth.

[14] Cohen B., Cowie A., Babiker M., Leip A., Smith P. (2021). Co-benefits and trade-offs of climate change mitigation actions and the Sustainable Development Goals. Sustain. Prod. Consum..

[15] (2021). Reinventing coal country: Reclaiming America's abandoned mine lands.

[16] Fennell A.-M. (2020). Mining on Federal Lands: More Than 800 Operations Authorized to Mine and Total Mineral Production Is Unknown. https://www.gao.gov/products/gao-20-461r.

[17] Keenan J., Holcombe S. (2021). Mining as a temporary land use: A global stocktake of post-mining transitions and repurposing. Extr. Ind. Soc..

[18] Zanini M.T.F., Migueles C.P., Gambirage C., Silva J. (2023). Barriers to local community participation in mining projects: The eroding role of power imbalance and information asymmetry. Resour. Policy.

[19] Priya A.K., Muruganandam M., Ali S.S., Kornaros M. (2023). Clean-Up of Heavy Metals from Contaminated Soil by Phytoremediation: A Multidisciplinary and Eco-Friendly Approach. Toxics.

[20] Angulo-Bejarano P.I., Puente-Rivera J., Cruz-Ortega R. (2021). Metal and Metalloid Toxicity in Plants: An Overview on Molecular Aspects. Plants.

[21] Wahab A., Muhammad M., Munir A., Abdi G., Zaman W., Ayaz A., Khizar C., Reddy S.P.P. (2023). Role of Arbuscular Mycorrhizal Fungi in Regulating Growth, Enhancing Productivity, and Potentially Influencing Ecosystems under Abiotic and Biotic Stresses. Plants.

[22] Li G., Yan L., Chen X., Lam S.S., Rinklebe J., Yu Q., Yang Y., Peng W., Sonne C. (2023). Phytoremediation of cadmium from soil, air and water. Chemosphere.

[23] Chen M., Guo L., Ramakrishnan M., Fei Z., Vinod K.K., Ding Y., Jiao C., Gao Z., Zha R., Wang C. (2022). Rapid growth of Moso bamboo (Phyllostachys edulis): Cellular roadmaps, transcriptome dynamics, and environmental factors. Plant Cell.

[24] Guo Z.H., Ma P.F., Yang G.Q., Hu J.Y., Liu Y.L., Xia E.H., Zhong M.C., Zhao L., Sun G.L., Xu Y.X. (2019). Genome Sequences Provide Insights into the Reticulate Origin and Unique Traits of Woody Bamboos. Mol. Plant.

[25] Patel B., Patel A., Syed B.A., Gami B., Patel P. (2021). Assessing economic feasibility of bio-energy feedstock cultivation on marginal lands. Biomass Bioenergy.

[26] Bian F., Zhong Z., Zhang X., Yang C., Gai X. (2020). Bamboo – An untapped plant resource for the phytoremediation of heavy metal contaminated soils. Chemosphere.

[27] Savage, E. (2021). Repairing the Damage: The costs of delaying reclamation at modern-era mines.

[28] Mzimela H.M.M., Izegaegbe J.I. (2021). Metal behaviour in water, sediment and fish from the St Lucia system: implications for public health and ecosystem impact. Environ. Monit. Assess..

[29] Núñez J.V., Pineda A.S., Pérez J.V., Zachrisson I.R. (2021). Assessment of Heavy Metal Concentration in Water, Soils and Sediments of La Villa River Basin, Panama. Recent Progress in Plant and Soil Research.

[30] Zunaidi A.A., Lim L.H., Metali F. (2021). Transfer of heavy metals from soils to curly mustard (Brassica juncea (L.) Czern.) grown in an agricultural farm in Brunei Darussalam. Heliyon.

[31] Samuel B., D Timothy O.T., Olayinka O., Olusegun O., Emmanuel B. (2020). Evaluation of the Impacts of Metals on Soil Samples, Serum Creatinine and Blood Urea Nitrogen of Residents in Selected Industrial Communities in a Developing Country. Env. cont. Reviews.

[32] Lufthansa U., Titah H., Pratikno H. (2021). The Ability of Mangrove Plant on Lead Phytoremediation at Wonorejo Estuary, Surabaya, Indonesia. J. Ecol. Eng..

[33] Akers D.B., MacCarthy M.F., Cunningham J.A., Annis J., Mihelcic J.R. (2015). Lead (Pb) Contamination of Self-Supply Groundwater Systems in Coastal Madagascar and Predictions of Blood Lead Levels in Exposed Children. Environ. Sci. Technol..

[34] Chheang L., Thongkon N., Sriwiriyarat T., Thanasupsin S.P. (2021). Heavy Metal Contamination and Human Health Implications in the Chan Thnal Reservoir, Cambodia. Sustainability.

[35] Silva R.B.P.d., Campos M.C.C., Silva L.S., Filho E.G.d.B., Lima A.F.L.d., Pinheiro E.N., Cunha J.M. (2020). Concentration of Heavy Metals in Soils under Cemetery Occupation in Amazonas, Brazil. Soil Sediment Contam.: Int. J..

[36] Melila M., Rajendran R., Lumo A.K., Arumugam G., Kpemissi M., Sadikou A., Lazar G., Amouzou K. (2019). Cardiovascular dysfunction and oxidative stress following human contamination by fluoride along with environmental xenobiotics (Cd & Pb) in the phosphate treatment area of Togo, West Africa. J. Trace Elem. Med. Biol..

[37] Lancellotti B.V., Hensley D.A., Stryker R. (2023). Detection of heavy metals and VOCs in streambed sediment indicates anthropogenic impact on intermittent streams of the U.S. Virgin Islands. Sci. Rep..

[38] Abdul Hamid F.A.Z., Abu Bakar A.F., Ng T.F., Ghani A.A., Mohamad Zulkifley M.T. (2019). Distribution and contamination assessment of potentially harmful elements (As, Pb, Ni, Cd) in topsoil of Penang Island, Malaysia. Environ. Earth Sci..

[39] Pombo L., Teherán A., Piñeros Ricardo L., Celis C., Andrade Barreiro W.A., Rodriguez A O.E. (2020). Contamination of staple crops by heavy metals in Sibaté, Colombia. Heliyon.

[40] Gottesfeld P., Were F.H., Adogame L., Gharbi S., San D., Nota M.M., Kuepouo G. (2018). Soil contamination from lead battery manufacturing and recycling in seven African countries. Environ. Res..

[41] Arya S., Rautela R., Chavan D., Kumar S. (2021). Evaluation of soil contamination due to crude E-waste recycling activities in the capital city of India. Process Saf. Environ. Protect..

[42] Cruzado-Tafur E., Torró L., Bierla K., Szpunar J., Tauler E. (2021). Heavy metal contents in soils and native flora inventory at mining environmental liabilities in the Peruvian Andes. J. S. Am. Earth Sci..

[43] Kumar S., Rahman M.A., Islam M.R., Hashem M.A., Rahman M.M. (2022). Lead and other elements-based pollution in soil, crops and water near a lead-acid battery recycling factory in Bangladesh. Chemosphere.

[44] Rodríguez-Hernández A., Lázaro I., Razo I., Briones-Gallardo R. (2021). Geochemical and mineralogical characterization of stream sediments impacted by mine wastes containing arsenic, cadmium and lead in North-Central Mexico. J. Geochem. Explor..

[45] Kfle G., Asgedom G., Goje T., Abbebe F., Habtom L., Hanes H. (2020). The Level of Heavy Metal Contamination in Selected Vegetables and Animal Feed Grasses Grown in Wastewater Irrigated Area, around Asmara, Eritrea. J. Chem..

[46] Turner S., Graham E., Macphail R., Duncan L., Rose N.L., Yang H., Whittet R., Rosique-Esplugas C. (2021). Mercury enrichment in anthrosols and adjacent coastal sediments at a Classic Maya site, Marco Gonzalez, Belize. Geoarchaeology.

[47] Ramírez A., García G., Werner O., Navarro-Pedreño J., Ros R.M. (2021). Implications of the Phytoremediation of Heavy Metal Contamination of Soils and Wild Plants in the Industrial Area of Haina, Dominican Republic. Sustainability.

[48] Vinha Guerreiro Silva M.M., Silva Cabral-Pinto M.M., Dinis P. (2021). Geochemistry of subtropical arenosols from Kuito region (Angola). Urbanization effects and environmental implications. J. Afr. Earth Sci..

[49] Umeobi E.C., Azuka C.V., Ofem K.I., John K., Nemeček K., Jidere C.M., Ezeaku P.I. (2024). Evaluation of potentially toxic elements in soils developed on limestone and lead-zinc mine sites in parts of southeastern Nigeria. Heliyon.

[50] Mbodji M., Baskali-Bouregaa N., Barbier-Bessueille F., Ayouni-Derouiche L., Diop C., Fall M., Gilon N. (2022). Speciation of metals by sequential extractions of agricultural soils located near a dumpsite for prediction of element availability to vegetables. Talanta.

[51] Fifi U., Winiarski T., Emmanuel E. (2013). Assessing the Mobility of Lead, Copper and Cadmium in a Calcareous Soil of Port-au-Prince, Haiti. Int. J. Environ. Res. Publ. Health.

[52] Yadav I.C., Devi N.L., Singh V.K., Li J., Zhang G. (2019). Spatial distribution, source analysis, and health risk assessment of heavy metals contamination in house dust and surface soil from four major cities of Nepal. Chemosphere.

[53] Wongsasuluk P., Tun A.Z., Chotpantarat S., Siriwong W. (2021). Related health risk assessment of exposure to arsenic and some heavy metals in gold mines in Banmauk Township, Myanmar. Sci. Rep..

[54] Tran T.S., Dinh V.C., Nguyen T.A.H., Kim K.-W. (2022). Soil contamination and health risk assessment from heavy metals exposure near mining area in Bac Kan province, Vietnam. Environ. Geochem. Health.

[55] Romero-Mujalli G., Melendez W. (2023). Nutrients and trace elements of semi-arid dwarf and fully developed mangrove soils, northwestern Venezuela. Environ. Earth Sci..

[56] Kříbek B., Nyambe I., Majer V., Knésl I., Mihaljevič M., Ettler V., Vaněk A., Penížek V., Sracek O. (2019). Soil contamination near the Kabwe Pb-Zn smelter in Zambia: Environmental impacts and remediation measures proposal. J. Geochem. Explor..

[57] Amphalop N., Suwantarat N., Prueksasit T., Yachusri C., Srithongouthai S. (2020). Ecological risk assessment of arsenic, cadmium, copper, and lead contamination in soil in e-waste separating household area, Buriram province, Thailand. Environ. Sci. Pollut. Res. Int..

[58] Muimba-Kankolongo A., Banza Lubaba Nkulu C., Mwitwa J., Kampemba F.M., Mulele Nabuyanda M., Haufroid V., Smolders E., Nemery B. (2021). Contamination of water and food crops by trace elements in the African Copperbelt: A collaborative cross-border study in Zambia and the Democratic Republic of Congo. Environmental Advances.

[59] Sosa D., Hilber I., Buerge-Weirich D., Faure R., Escobar A., Bucheli T.D. (2022). Heavy metals in soils of Mayabeque, Cuba: multifaceted and hardly discernable contributions from pedogenic and anthropogenic sources. Environ. Monit. Assess..

[60] Jones D.H., Yu X., Guo Q., Duan X., Jia C. (2022). Racial Disparities in the Heavy Metal Contamination of Urban Soil in the Southeastern United States. Int. J. Environ. Res. Publ. Health.

[61] Rosado Rodríguez G., Otero Morales E. (2020). Assessment of heavy metal contamination at Tallaboa Bay (Puerto Rico) by marine sponges' bioaccumulation and fungal community composition. Mar. Pollut. Bull..

[62] Iaquinta F., Antelo E., Machado I. (2023). Distribution of Inorganic Contaminants Along the Coast of Ciudad de la Costa, Uruguay. Arch. Environ. Contam. Toxicol..

[63] Vongdala N., Tran H.D., Xuan T.D., Teschke R., Khanh T.D. (2018). Heavy Metal Accumulation in Water, Soil, and Plants of Municipal Solid Waste Landfill in Vientiane, Laos. Int. J. Environ. Res. Publ. Health.

[64] Puri S.B., Rao B.K.R. (2023). Ecotoxicological risks of metals in the subsistence food garden soils of Watut River floodplains, Papua New Guinea. Environ. Geochem. Health.

[65] Hirwa H., Nshimiyimana F.X., Ngendahayo E., Akimpaye B., Nahayo L., Ngamata O.M., de Dieu Bazimenyera J. (2019). Evaluation of Soil Contamination in Mining Areas of Rwanda. American Journal of Water Science and Engineering.

[66] Nuwamanya E., Byamugisha D., Nakiguli C.K., Angiro C., Khanakwa A.V., Omara T., Ocakacon S., Onen P., Omoding D., Opio B. (2024). Exposure and Health Risks Posed by Potentially Toxic Elements in Soils of Metal Fabrication Workshops in Mbarara City, Uganda. J. Xenobiot..

[67] Ochoa M., Tierra W., Tupuna-Yerovi D.S., Guanoluisa D., Otero X.L., Ruales J. (2020). Assessment of cadmium and lead contamination in rice farming soils and rice (Oryza sativa L.) from Guayas province in Ecuador. Environ. Pollut..

[68] Cooke C.A., Curtis J.H., Kenney W.F., Drevnick P., Siegel P.E. (2022). Caribbean Lead and Mercury Pollution Archived in a Crater Lake. Environ. Sci. Technol..

[69] Berihun B.T., Amare D.E., Raju R.P., Ayele D.T., Dagne H. (2021). Determination of the Level of Metallic Contamination in Irrigation Vegetables, the Soil, and the Water in Gondar City, Ethiopia. Nutr. Diet. Suppl..

[70] Abbass O.A.E., Elhassan A.M., Abdelgadir A.E. (2023). Detection of Heavy Metals Concentrations in Agriculture Plants Near Landfills: Case Study in Wadafiea, Sudan. International Journal of Computational Methods and Experimental Measurements.

[71] Kroonenberg S., Wong T., Bijnaar G., Finkie R., Goenopawiro K., Asneel S., Lin-Tsung M., Nanan R., Ramdas K., Sitaram P. (2022). Mercury background values in soils and saprolites in the gold-rich greenstone belt of Suriname, Guiana Shield: The role of parent rock and residual enrichment. Sci. Total Environ..

[72] Mahdi Ahmed M., Doumenq P., Awaleh M.O., Syakti A.D., Asia L., Chiron S. (2017). Levels and sources of heavy metals and PAHs in sediment of Djibouti-city (Republic of Djibouti). Mar. Pollut. Bull..

[73] Hernández E., Obrist-Farner J., Brenner M., Kenney W.F., Curtis J.H., Duarte E. (2020). Natural and anthropogenic sources of lead, zinc, and nickel in sediments of Lake Izabal, Guatemala. J. Environ. Sci..

[74] Wells E.C., Waters C.K., Tricarico A.R., Fox G.L. (2017). Agroindustrial Soilscapes in the Caribbean: A Geochemical Perspective from Betty’s Hope, Antigua. Environ. Archaeol..

[75] Marove C.A., Sotozono R., Tangviroon P., Tabelin C.B., Igarashi T. (2022). Assessment of soil, sediment and water contaminations around open-pit coal mines in Moatize, Tete province, Mozambique. Environmental Advances.

[76] Manirakiza N., Ndikumana T., Jung C.G. (2020). Heavy metals impacted soils from dumped municipal solid waste in Buterere - Burundi: Health risk assessment. Int. J. Innovat. Appl. Stud..

[77] Anglin-Brown B., Armour-Brown A., Lalor G.C., Preston J., Vutchkov M.K. (1996). Lead in a residential environment in Jamaica. Environ. Geochem. Health.

[78] Michel J., Zengel S. (1998). Monitoring of oysters and sediments in Acajutla, El Salvador. Mar. Pollut. Bull..

[79] Rai R., Sharma S., Gurung D.B., Sitaula B.K. (2019). Heavy metal contamination in sediments from vehicle washing: a case study of Olarong Chhu Stream and Paa Chhu River, Bhutan. Int. J. Environ. Stud..

[80] Kamara M.A. (2019).

[81] Soumahoro N.S., Kouassi N.L.B., Yao K.M., Kwa-Koffi E.K., Kouassi A.M., Trokourey A. (2021). Impact of municipal solid waste dumpsites on trace metal contamination levels in the surrounding area: a case study in West Africa, Abidjan, Cote d’Ivoire. Environ. Sci. Pollut. Res. Int..

[82] Marcantonio R.A., Field S.P., Sesay P.B., Lamberti G.A. (2021). Identifying human health risks from precious metal mining in Sierra Leone. Reg. Environ. Change.

[83] Wang Y., Tan S.N., Mohd Yusof M.L., Ghosh S., Lam Y.M. (2022). Assessment of heavy metal and metalloid levels and screening potential of tropical plant species for phytoremediation in Singapore. Environ. Pollut..

[84] Mlangeni A.T., Chinthenga E., Kapito N.J., Namaumbo S., Feldmann J., Raab A. (2023). Safety of African grown rice: Comparative review of As, Cd, and Pb contamination in African rice and paddy fields. Heliyon.

[85] Makokha A., Mghweno L., Magoha H., Nakajugo A., Wekesa J., Jkuat, and Kenya (2008). Environmental lead pollution and contamination in food aroud Lake Victoria, Kisumu, Kenya. Afr. J. Environ. Sci. Technol..

[86] Bongoua-Devisme A., Balland-Bolou-Bi C., Koffi K., Bolou B., D G., Donatien K., Adiaffi B., Albert Y.-K., Djagoua E., Valère M.m. (2018). Assessment of heavy metal contamination degree of municipal open-air dumpsite on surrounding soils: Case of dumpsite of Bonoua, Ivory Coast. Int. J. Eng. Res. Gen. Sci..

[87] Binh N.T.L., Hoang N.T., Truc N.T.T., Khang V.D., Le H.A. (2021). Estimating the Possibility of Lead Contamination in Soil Surface due to Lead Deposition in Atmosphere. J. Nanomater..

[88] Kaonga C.C., Kosamu I.B., Lakudzala D.D., Mbewe R., Thole B., Monjerezi M., Chidya R.C.G., Kuyeli S., Mkali S., Sajidu S.M.I. (2017). A review of heavy metals in soil and aquatic systems of urban and semi-urban areas in Malawi with comparisons to other selected countries. Afr. J. Environ. Sci. Technol..

[89] Schwabe S.J., Cathcart E.M., Carew J.L. (2010). Proceedings of the 14th Symposium on Geology of the Bahamas and Other Carbonate Regions.

[90] Rojas-Conejo J., Pavón F.P., Serrano A.S., Gestel C.A.M.v., Benavides C.G., Sanabria G.D. (2021). Mining environmental liabilities: a potential source of metal contamination for freshwater ecosystems in Costa Rica. Geographical Journal of Central America.

[91] Ona L.F. (2010). Lead (Pb) contamination of dust from schools in an urbanized city in the Philippines. Int. J. Environ. Sustain Dev..

[92] Taylor M.P., Isley C.F., Fry K.L., Liu X., Gillings M.M., Rouillon M., Soltani N.S., Gore D.B., Filippelli G.M. (2021). A citizen science approach to identifying trace metal contamination risks in urban gardens. Environ. Int..

[93] Tapia-Gatica J., Selles I., Bravo M.A., Tessini C., Barros-Parada W., Novoselov A., Neaman A. (2022). Global issues in setting legal limits on soil metal contamination: A case study of Chile. Chemosphere.

[94] López J.E., Marín J.F., Saldarriaga J.F. (2024). Assessing pollution degree and human health risks from hazardous element distribution in soils near gold mines in a Colombian Andean region: Correlation with phytotoxicity biomarkers. Chemosphere.

[95] Bundschuh J., Schneider J., Alam M.A., Niazi N.K., Herath I., Parvez F., Tomaszewska B., Guilherme L.R.G., Maity J.P., López D.L. (2021). Seven potential sources of arsenic pollution in Latin America and their environmental and health impacts. Sci. Total Environ..

[96] Coronel-Teixeira R., Cañiza B., Fretes J., Rodríguez M., Pasten M., Escurra C.M., Pérez-Bejarano D. (2022). Relevant aspects on biomonitoring of heavy metal concentration in environmental air in Asunción city. Revista científica ciencias de la salud.

[97] Kao C.S., Wang Y.L., Chuang T.W., Jiang C.B., Hsi H.C., Liao K.W., Chien L.C. (2021). Effects of soil lead exposure and land use characteristics on neurodevelopment among children under 3 years of age in northern Taiwan. Environ. Pollut..

[98] Perera W.P.R.T., Dayananda M.D.N.R., Dissanayake D.M.U.C., Rathnasekara R.A.S.D., Botheju W.S.M., Liyanage J.A., Weragoda S.K., Kularathne K.A.M. (2021). Risk Assessment of Trace Element Contamination in Drinking Water and Agricultural Soil: A Study in Selected Chronic Kidney Disease of Unknown Etiology (CKDu) Endemic Areas in Sri Lanka. J. Chem..

[99] Mununga Katebe F., Raulier P., Colinet G., Ngoy Shutcha M., Mpundu Mubemba M., Jijakli M.H. (2023). Assessment of Heavy Metal Pollution of Agricultural Soil, Irrigation Water, and Vegetables in and Nearby the Cupriferous City of Lubumbashi, (Democratic Republic of the Congo). Agronomy.

[100] Mudzengi B.K. (2018). The spatial distribution of soil lead pollution in the Middle Mukuvisi Catchment, Harare, Zimbabwe. Braz. J. Biol. Sci..

[101] Fuke P., Kumar M., Sawarkar A.D., Pandey A., Singh L. (2021). Role of microbial diversity to influence the growth and environmental remediation capacity of bamboo: A review. Ind. Crop. Prod..

[102] Richard Barret, J.G., M.J. Williams (2021). Giant Cane and Other Native Bamboos: Establishment and Use for Conservation of Natural Resources in the Southeast.

[103] Ahmad Z., Upadhyay A., Ding Y., Emamverdian A., Shahzad A., Ahmad Z., Ding Y., Shahzad A. (2021). Biotechnological Advances in Bamboo: The “Green Gold” on the Earth.

[104] Remadevi O.K., Kaur S., Batish D.R., Singh H.P., Kohli R. (2022). Biodiversity in India: Status, Issues and Challenges.

[105] Dorairaj D., Osman N. (2021). Present practices and emerging opportunities in bioengineering for slope stabilization in Malaysia: An overview. PeerJ.

[106] Nfornkah B.N., Nath A.J., Kaam R., Chimi C.D., Mezafack K.L., Palombini F.L., Nogueira F.M. (2023). Bamboo Science and Technology.

[107] Ekwe N.B., Tyufekchiev M.V., Salifu A.A., Schmidt-Rohr K., Zheng Z., Maag A.R., Tompsett G.A., Cai C.M., Onche E.O., Ates A. (2022). Bamboo as a Cost-Effective Source of Renewable Carbon for Sustainable Economic Development in Low- and Middle-Income Economies. Energies.

[108] Gul E., Al Bkoor Alrawashdeh K., Masek O., Skreiberg Ø., Corona A., Zampilli M., Wang L., Samaras P., Yang Q., Zhou H. (2021). Production and use of biochar from lignin and lignin-rich residues (such as digestate and olive stones) for wastewater treatment. J. Anal. Appl. Pyrol..

[109] Yadav M., Mathur A. (2021). Bamboo as a sustainable material in the construction industry: An overview. Mater. Today: Proc..

[110] Zheng Z., Yan N., Lou Z., Jiang X., Zhang X., Chen S., Xu R., Liu C., Xu L. (2023). Modification and Application of Bamboo-Based Materials: A Review—Part I: Modification Methods and Mechanisms. Forests.

[111] Yu L., Pei J., Zhao Y., Wang S. (2021). Physiological changes of bamboo (fargesia yunnanensis) shoots during storage and the related cold storage mechanisms. Front. Plant Sci..

[112] Yang Y., Yu S.Y., Zhu Y., Shao J. (2014). The Making of Fired Clay Bricks in China Some 5000 Years Ago. Archaeometry.

[113] Emamverdian A., Ding Y., Ranaei F., Ahmad Z. (2020). Application of Bamboo Plants in Nine Aspects. Sci. World J..

[114] Amoah P., Eweje G. (2023). Examining the social sustainability strategies of multinational mining companies in a developing country. Soc. Responsib. J..

[115] (2021). Indigenous Fire Practices Shape Our Land.

[116] Dinara Ermakova J.W.B., Wainwright H., Vujic J. (2022).

[117] Weber, J., Forren, K., McCoy, S., and Black, K.J. (2022). The State of Abandoned Mine Reclamation: Perspectives from Pennsylvania Stakeholders.

[118] White B., Doole G.J., Pannell D.J., Florec V. (2012). Optimal environmental policy design for mine rehabilitation and pollution with a risk of non-compliance owing to firm insolvency. Aust. J. Agric. Resour. Econ..

[119] Méndez C., Simpson N., Johnson F. (2023). Climate Change 2023: Synthesis Report (Full Volume) Contribution of Working Groups I, II and III to the Sixth Assessment Report of the Intergovernmental Panel on Climate Change.

[120] Xu X., Xu P., Zhu J., Li H., Xiong Z. (2022). Bamboo construction materials:Carbon storage and potential to reduce associated CO2 emissions. Sci. Total Environ..

[121] Gupta S., Kashani A., Mahmood A.H., Han T. (2021). Carbon sequestration in cementitious composites using biochar and fly ash – Effect on mechanical and durability properties. Construct. Build. Mater..

[122] Mohan D., Abhishek K., Sarswat A., Patel M., Singh P., Pittman C.U. (2018). Biochar production and applications in soil fertility and carbon sequestration – a sustainable solution to crop-residue burning in India. RSC Adv..

[123] Xu H., Wang Z., Huang J., Jiang Y. (2021). Thermal Catalytic Conversion of Biomass-Derived Glucose to Fine Chemicals. Energy Fuels.

[124] Huang C., Fang G., Yu L., Zhou Y., Meng X., Deng Y., Shen K., Ragauskas A.J. (2020). Maximizing enzymatic hydrolysis efficiency of bamboo with a mild ethanol-assistant alkaline peroxide pretreatment. Bioresour. Technol..

[125] Barot S. (2022). Renewable Energy for Sustainable Growth Assessment.

[126] Lee U., Kwon H., Wu M., Wang M. (2021). Retrospective analysis of the U.S. corn ethanol industry for 2005–2019:implications for greenhouse gas emission reductions. Biofuel. Bioprod. Biorefin..

[127] Wietschel L., Messmann L., Thorenz A., Tuma A. (2021). Environmental benefits of large-scale second-generation bioethanol production in the EU: An integrated supply chain network optimization and life cycle assessment approach. J. Ind. Ecol..

[128] Liu F., Short M.D., Alvarez-Gaitan J.P., Guo X., Duan J., Saint C., Chen G., Hou L. (2020). Environmental life cycle assessment of lignocellulosic ethanol-blended fuels: A case study. J. Clean. Prod..

[129] Andersson Ö., Börjesson P. (2021). The greenhouse gas emissions of an electrified vehicle combined with renewable fuels: Life cycle assessment and policy implications. Appl. Energy.

[130] Gandam P.K., Chinta M.L., Pabbathi N.P.P., Baadhe R.R., Sharma M., Thakur V.K., Sharma G.D., Ranjitha J., Gupta V.K. (2022). Second-generation bioethanol production from corncob – A comprehensive review on pretreatment and bioconversion strategies, including techno-economic and lifecycle perspective. Ind. Crop. Prod..

[131] Rathour R., Kumar H., Prasad K., Anerao P., Kumar M., Kapley A., Pandey A., Kumar Awasthi M., Singh L. (2022). Multifunctional applications of bamboo crop beyond environmental management: an Indian prospective. Bioengineered.

[132] Thomas G., Sheridan C., Holm P.E. (2022). A critical review of phytoremediation for acid mine drainage-impacted environments. Sci. Total Environ..

[133] Akoto O., Yakubu S., Ofori L.A., Bortey-Sam N., Boadi N.O., Horgah J., Sackey L.N.A. (2023). Multivariate studies and heavy metal pollution in soil from gold mining area. Heliyon.

[134] Rouhani A., Skousen J., Tack F.M.G. (2023). An Overview of Soil Pollution and Remediation Strategies in Coal Mining Regions. Minerals.

[135] Rajendran S., Priya T.A.K., Khoo K.S., Hoang T.K.A., Ng H.-S., Munawaroh H.S.H., Karaman C., Orooji Y., Show P.L. (2022). A critical review on various remediation approaches for heavy metal contaminants removal from contaminated soils. Chemosphere.

